# Understanding the policy dynamics of COVID-19 vaccination in Ghana through the lens of a policy analytical framework

**DOI:** 10.1186/s12961-022-00896-1

**Published:** 2022-09-01

**Authors:** Roger A. Atinga, Augustina Koduah, Gilbert Abotisem Abiiro

**Affiliations:** 1 Department of Public Administration and Health Services Management, University of Ghana Business School, P.O. Box LG 78, Legon, Accra, Ghana; 2 Department of Pharmacy Practice and Clinical Pharmacy, School of Pharmacy, College of Health Sciences, University of Ghana, P. O. Box LG 43, Legon, Accra, Ghana; 3 Department of Health Services, Policy, Planning, Management and Economics, School of Public Health, University for Development Studies, P.O. Box 1883, Tamale, Ghana; 4 Department of Population and Reproductive Health, School of Public Health, University for Development Studies, P. O. Box 1883, Tamale, Ghana

**Keywords:** COVID-19, Vaccination, Policy analysis, Political, Policy framing, Issue characteristics, Actor power, Ghana

## Abstract

**Background:**

Ghana became the first African country to take delivery of the first wave of the AstraZeneca/Oxford vaccine from the COVAX facility. But why has this promising start of the vaccination rollout not translated into an accelerated full vaccination of the population? To answer this question, we drew on the tenets of a policy analytical framework and analysed the diverse interpretations, issue characteristics, actor power dynamics and political context of the COVID-19 vaccination process in Ghana.

**Methods:**

We conducted a rapid online review of media reports, journal articles and other documents on debates and discussions of issues related to framing of the vaccination rollout, social constructions generated around vaccines, stakeholder power dynamics and political contentions linked to the vaccination rollout. These were complemented by desk reviews of parliamentary reports.

**Results:**

The COVID-19 vaccination was mainly framed along the lines of public health, gender-centredness and universal health coverage. Vaccine acquisition and procurement were riddled with politics between the ruling government and the largest main opposition party. While the latter persistently blamed the former for engaging in political rhetoric rather than a tactical response to vaccine supply issues, the former attributed vaccine shortages to vaccine nationalism that crowded out fair distribution. The government’s efforts to increase vaccination coverage to target levels were stifled when a deal with a private supplier to procure 3.4 million doses of the Sputnik V vaccine collapsed due to procurement breaches. Amidst the vaccine scarcity, the government developed a working proposal to produce vaccines locally which attracted considerable interest among pharmaceutical manufacturers, political constituents and donor partners. Regarding issue characteristics of the vaccination, hesitancy for vaccination linked to misperceptions of vaccine safety provoked politically led vaccination campaigns to induce vaccine acceptance.

**Conclusions:**

Scaling up vaccination requires political unity, cohesive frames, management of stakeholder interests and influence, and tackling contextual factors promoting vaccination hesitancy.

## Background

The fight against the severe acute respiratory syndrome coronavirus 2 (SARS-CoV-2), responsible for the coronavirus disease 2019 (COVID-19) pandemic, is gradually taking shape but is far from being won [1]. Various countries have now taken delivery of the COVID-19 vaccines after months of investment in scientific research and series of international diplomatic engagements that culminated in the COVID-19 Vaccines Global Access (COVAX) facility. The COVAX facility is an initiative operated by WHO, Gavi, the Vaccine Alliance, and the Coalition for Epidemic Preparedness Innovations (CEPI) for a fair and equitable supply of vaccines globally [2]. As of May 2022, the share of the world’s population that had received at least one dose of the COVID-19 vaccines was 65.8%, with low- and middle-income countries (LMICs) accounting for 16.2%, in contrast with more than 80% in high-income countries [3]. The scientific discovery, production and distribution of COVID-19 vaccines have been intertwined with politics [4] and policy twists and turns [5]. Vaccine manufacturers endured enormous political pressure from countries and influential individual political elites to speed up production to break the cycle of COVID-19 transmission [5]. Even before the final stage of human trials and regulatory approvals of the vaccines, politics took centre stage in the global value chain system in what became known as vaccine nationalism [6].

Relying on their global power, influence and resource strength, some of the rich countries such as the United States and countries within the European Union (EU) went out of their way and directly signed bilateral pre-purchase agreements with vaccine manufacturers to take early delivery of vaccines [6], contrary to international agreement to channel all COVID-19-related vaccines to the COVAX facility for fair distribution [7]. As a result, there has been gross inequity and poor access to vaccines in LMICs, as 10 wealthy countries have procured and administered more than 75% of vaccines produced so far [7]. Additionally, the distribution policies of the vaccines are determined by the developed countries. Most countries, especially LMICs, only determine in-country receipt and mechanisms of vaccination rollouts. While some countries have been proactive in implementing policies that prioritize early vaccination of health workers and high-risk populations [8], others are less responsive because of underlying negative beliefs, scepticism and misinformation about the COVID-19 vaccines [8].

On 24 February 2021 Ghana became the first country in Africa to take delivery of the first wave of the AstraZeneca/Oxford vaccine shipped via the COVAX facility [9]. This historic milestone in the global rollout of the COVID-19 vaccines attracted waves of questions about the level of power and political influence of the Ghanaian government on the global political arena. Such questions were partly answered by the high political commitment and policy levers initiated by the Government of Ghana to combat COVID-19. The President of Ghana, in announcing a partial lockdown of the country in March 2020 to mitigate COVID-19 transmission, made a profound statement: “*We know how to bring the economy back to life. What we do not know is how to bring people back to life*” [10]. The President also made more than 20 live televised speeches between March 2020 and June 2021 on the state of COVID-19 in Ghana, constantly highlighting caution, responsible behaviours and strict adherence to protocols before the arrival of vaccines. In December 2020, soon after a national political election, the President in a speech assured Ghanaians of the government’s efforts to secure vaccines in a timely manner, noting that “*Ghana will not be left behind in having access to the vaccines*” [11]. Taken together, such speeches not only gained widespread international attention but also fit with the broader scheme of politicization of the pandemic and the vaccine rollout process [12].

The arrival of the first batch of vaccines and the start of the vaccination process were subjects of internal political contention and concerns about vaccine availability, acquisition and distribution, and inclusive vaccination of the poor and the vulnerable. These political manifestations of the vaccination rollout were partly the antecedent of the level of political language, expression, debates and frames used to portray the COVID-19 and its mitigation [13, 14]. Alongside politics, the vaccination rollout attracted diverse interpretations, interest and issue characteristics that adversely shaped attitudes towards vaccination [15]. There are marked regional variations in the uptake of vaccination and willingness to vaccinate, driven primarily by social, behavioural and environmental factors [16]. Efforts to expand vaccination coverage can be met with considerable challenges without a unified political direction and strategy, adequate management of stakeholder interests and efforts to tackle factors across socio-ecological levels shaping vaccine acceptance.

We drew on a policy analysis framework to inform an understanding of the political dynamics, stakeholder actions and interpretations, and contextual issues that shaped the COVID-19 vaccine rollout in Ghana. The COVID-19 vaccination is ongoing amid supply challenges, but the political machinations combined with divergent perspectives, interests, interpretations and behavioural elements that characterized the vaccination rollout need to be understood to maximize net gains of the vaccination. In addition, while health behaviours may be nonpartisan, politically, the rhetoric and actions of political constituents, their framing of COVID-19 vaccination and the strategies they adopt in the vaccination rollout can have implications for scaling up vaccination coverage. This study was conducted to understand why a promising start of the COVID-19 vaccination rollout has not translated into full vaccination coverage of the population. We specifically explored and analysed (1) the political and stakeholder framing of the COVID-19 vaccination, (2) the issue characteristics of the COVID-19 vaccination, (3) stakeholder power dynamics (interest, positions and influences) linked to the COVID-19 vaccination and (4) the political dynamics surrounding the COVID-19 vaccination.

## Methods

### Analytical framework

The study adopted the Shiffman and Smith [17] policy analytical framework to explore the policy and political dynamics of the COVID-19 vaccination rollout in Ghana. The framework has been widely used to explore how and why health policy issues or initiatives gain, retain or lose political attention [18–21]. According to the framework, the level of attention given to a health issue is determined by the ideas used to frame it, the characteristics of the issue itself, the actor power dynamics surrounding it and the political context within which the issue is being considered [17].

Guided by the framework, we analysed ongoing debates, discussions and publications related to the (1) framing of the vaccination rollout, (2) issue characteristics of the vaccination, (3) actor power dynamics (interest, positions and influences of actors connected to the vaccination) and (4) political behaviours and dynamics underlying the vaccination rollout. Framing was assessed by focusing on ideas used to give meaning to the vaccination process. Issue characteristics were determined by examining how the social and political environment shape perceptions about vaccines, attitude towards vaccination, vaccine campaign and the approach to vaccination. We identified all the internal and external stakeholders with roles connected to the COVID-19 vaccination process and assessed their interests/concerns, their positions and their influence on policy direction for vaccination [22, 23]. Attention was paid to alliances, mobilization and activities of actors that promote or undermine vaccination. Under political context, we assessed the involvement of political constituents, political elites and political institutions in the acquisition and administration of the COVID-19 vaccines. Bipartisan political pronouncements and debates about the vaccination were also assessed.

### Research approach, and data extraction and analysis 

We conducted a rapid review drawing on an interpretive synthesis of published and unpublished journal articles, grey literature, media and institutional reports between November 2020 and August 2021. A trained research assistant carried out the online search using Google Scholar, Google and Google Chrome search engines. The search terms were defined at three levels: level 1 included terms such as COVID-19, coronavirus, COVID and SARS-CoV-2; level 2 comprised terms describing vaccination including COVID-19 vaccines, COVID-19 vaccination, immunization, COVID-19 jab and COVAX; and level 3 consisted of terms denoting the context of COVID-19 vaccination in Ghana. The three authors independently and systematically cross-checked the online sources to validate the search materials generated by the research assistant. After 3 weeks of the search process, a total of 200 online materials were obtained and screened by the authors, leading to the retention of 120 materials generally reporting content relating to COVID-19 vaccination in Ghana. The retained materials were further assessed for their suitability and mapped against the objectives of the study. This led to the removal of mismatches, duplicates and content from unknown sources, and the retention of 54 materials distributed as follows: media reports (print and electronic: *n* = 22), press briefings (presidential, cabinet, political parties and interest groups: *n* = 10), reports (including minutes of meetings: *n* = 7) and journal articles (*n* = 14). We also hand-searched the Parliament of Ghana’s library for transcripts of parliamentary debates and proceedings between January and May 2021 (*n* = 4). In all, 57 items were used for the analysis.

The data were analysed partly manually and partly aided by NVivo 12 computer-assisted qualitative data analytical software. The online materials were exported to the software for coding and thematic analysis. Printed documents were manually coded. The analysis was guided by the Hsieh and Shannon [24] thematic analysis framework. The main items in the policy analytical framework of the study formed initial broad deductive codes/themes. Emerging subthemes were derived through progressive inductive coding of the materials (Table 1). All three authors independently coded the material to enhance inter-coder reliability.

**Table 1:** Summary of the results of the thematic analysis

Major themes	Subthemes	Sources
1. Stakeholders’ framings of COVID-19 vaccination		
Vaccination as a public health issue	• Vaccination mitigates transmission of the virus • Vaccination minimizes COVID-19 morbidity and mortality • Vaccination halts effects of COVID-19 on health and well-being • Public health dimension of inoculation policy • Targeting the aged, vulnerable and those with comorbidities in the vaccination rollout	[25–27]
Gender-centred frame of vaccination	• Prioritizing women in the vaccination rollout • Giving women greater roles in the vaccination rollout • Targeting women to eliminate vulnerability to health risk	[25, 28, 29]
Vaccination as a universal health coverage issue	• Making vaccines available to everyone • Vaccination as a human right • Equitable coverage of vaccination • Redistributive tax (COVID-19 Health Recovery Levy) to finance vaccine needs of all individuals	[30–33]
2. Issue characteristics of COVID-19 vaccination		
Vaccine safety and hesitancy	• Misinformation about vaccine safety • Doubts regarding vaccine efficacy • Distrust in COVID-19 vaccines • Health workers’ hesitancy to get vaccinated • Public hesitancy towards vaccination	[8, 15, 34–41]
Delay in the second jab and change of the vaccination plan	• Irregular dosing intervals • Delays in receiving and administering the second jab • Extension of interval between first and second jab by 4 weeks • Reluctance to receive booster • Ghana Health Service assurance to the public of protection of the first jab	[16, 42, 43]
Politically led vaccination campaign	• President assurance to the public of vaccine safety • President and Vice-President received first jab on live television • Parliament advocated for inclusive vaccination • Members of Parliament to receive jabs in their constituencies to engender trust in the vaccines	[33, 39, 43–45]
3. Actor power dynamics surrounding COVID-19 vaccination		
Support for local production of vaccines	• President and Health Minister drove support for local vaccines • President set up committee to explore local production of vaccines • Political constituents supported local production of vaccines • Pharmaceutical Manufacturers Association of Ghana (PMAG) supported policy for local production of vaccines • Development partners supported local production of vaccines	[46–52]
4. Political context of COVID-19 vaccination		
The politics of vaccine availability	• President resolved to make vaccines available • Opposition political party criticized poor vaccine availability • Opposition party criticized government’s approach to vaccination • Vaccine nationalism affecting vaccine availability • Political pressure on government to secure more vaccines	[34, 42, 53–58]
The politics of vaccine procurement	• Pressure to secure vaccines led to procurement irregularities • Vaccines procured above ex-factory price • Government criticized for using middlemen to procure vaccines • Health Minister breached established procurement rules in vaccine procurement • Government justified high unit cost of doses • Health Minister spurned punitive measures	[32, 34, 35, 59, 60]

## Results

Table 1 summarizes results of the major themes and subthemes that emerged from the coding process. There were nine overarching themes and 42 subthemes derived from a range of literature sources. The findings are structured and presented in thematic order, supported by quotes extracted from the materials.

### Framing of the vaccination rollout

The main frames identified within the social and political spaces were vaccination as a public health issue, gender-centredness of vaccination and vaccination as a universal health coverage (UHC) issue.

#### COVID-19 vaccination as a public health issue

The COVID-19 pandemic and its mortality proportions are perceived by health stakeholders as a public health burden with a regressive impact on all aspects of socioeconomic growth [26]. Thus, political constituents, the general public and civil society organizations perceive the vaccination rollout as timely and relevant to mitigating transmission of the virus. In particular, the detection of the six highly contagious Delta variants in the country was a public health concern shifting attention from clinical interventions to responsive vaccination [25]. The public health frame also widely acknowledged the urgent need for vaccination to tackle the detrimental effects of COVID-19 on health and well-being.

During deliberations in the chamber of Parliament about COVID-19 vaccination in May 2021, a member of Parliament stated, “*We sit on a time bomb. Just look at COVID-19 and how this whole continent was found wanting and miserable and looking as beggars hoping that somebody would bring us vaccines*” [27]. Political framing of the vaccination rollout as a public health issue further reflected how the President consistently highlighted strict adherence to the COVID-19 protocols to minimize the burden of morbidity and mortality until vaccines were secured [40]. Even the framing of the inoculation policy considered the morbidity proportions of the pandemic in prioritizing persons with comorbidities, the aged (60+ years), the physically challenged and health workers among others in the first phase of the vaccination plan.

#### Gender-centred frame of vaccination

The gender-centred frame related to prioritizing women in the vaccination process as well as giving them a greater role in the vaccination rollout. The sentiment was that women are more vulnerable to health risks due to biological and social factors [28]. Gender norms within the Ghanaian context also place greater responsibility on women at the household level including child care. Targeting early vaccination of women was important to eliminate vulnerability to health risk among children and the household. Moreover, the pandemic disrupted access to quality maternal health services, leading to fear and anxiety among women in need of such services [25]. Consequently, channelling more vaccine resources and supplies to women was seen as crucial.

The history of women’s substantial contribution in eliminating the six killer diseases (measles, pertussis, diphtheria, tetanus, tuberculosis and poliomyelitis) was stressed as a reason that women should be at the epicentre of policies designed to roll out the vaccination exercise. This was further highlighted in Parliament on International Women’s Day, where members agreed that elements of vaccination campaign messages and communication should be targeted at women. For example, an honourable member of Parliament made a submission on the floor of the house: “*Much of the public education and sensitisation programmes should be targeted at women. Indeed, the experience of women to champion the vaccination agenda cannot be overemphasised. They have been at the forefront of the vaccination campaign in dealing with the six childhood killer diseases for years. It is imperative, therefore, for Ghana to leverage the power and influence of women to drive home the COVID-19 vaccination efforts*” [33].

#### Vaccination as a UHC issue

The UHC frame was concerned with the strategies adopted to make vaccines accessible at no cost in the medium to long term. This frame links COVID-19 vaccination to human rights and social protection as with other high-income countries [30]. The UHC vaccination agenda fits into the United Nations Human Rights Council framework tasking governments with adopting a pragmatic nondiscriminatory and universal human rights-based approach in administering COVID-19 vaccines [31]. Ghana’s Ministry of Health (MoH) COVID-19 inoculation policy somewhat draws on these human rights principles in prioritizing equity in the vaccination rollout. The policy provides for every Ghanaian to be vaccinated in the shortest possible time. The MoH further assured that “it will endeavour to secure vaccines for the Ghanaian people despite global shortages and cognizant of price and legal considerations” [32]. Apart from the goal of achieving equity and bridging the imbalanced distribution of vaccines, the government sought to deploy drones in delivering vaccines to hard-to-reach settings [61].

In keeping with the UHC agenda, the government introduced a COVID-19 Health Recovery Levy of 1% on the value of the taxable supply of goods and services and on the value of imports as part of a package to raise funds for COVID-19 mitigation including sustainable vaccine financing [33]. The tax levy was resisted by the opposition political parties arguing that Ghanaians were already overburdened with taxes and that any additional tax could significantly lower economic welfare among the majority outside the formal economy. But the position of the government was that the levy had a redistributive effect in that the rich were taxed to finance the vaccine needs of the poor. During the reading of the COVID-19 Health Recovery Levy Act in the chamber of Parliament, the Deputy Finance Minister highlighted the urgency of the bill, noting that “*some Ghanaians will be happy to be levied to buy vaccines to cover everybody in the country to eliminate the pandemic*” [33].

### Issue characteristics of the COVID-19 vaccination

Three main issue characteristics shaped the vaccination rollout identified in the literature: vaccine safety and hesitancy, revision of the inoculation plan and the politically led campaign for vaccination.

#### Vaccine safety and hesitancy for vaccination

The nature and scale of infodemic across multiple channels created widespread misperceptions regarding COVID-19 vaccines. The impression formed by a cross section of the population was that the vaccines were not safe for administration to human populations [8]. There were also reports that most communities were doubtful of the efficacy of the vaccines in preventing COVID-19 [34]. Fears that characterized misperceptions about vaccine safety were further heightened by rumours across the social media linking adverse blood coagulation events with the AstraZeneca vaccine. Consequently, the Ghana Food and Drugs Authority issued a statement confirming the clinical efficacy of the COVID-19 vaccines [62] and drawing the public attention to studies that confirmed the safety of the vaccines [36]. In spite of this, many Ghanaians adopted a cautious attitude towards the vaccines. A national-level study involving a sample of 2345 showed that about 41% of the respondents did not trust the clinical safety of COVID-19 vaccines in general [15].

The anxieties and uncertainties about the safety of the vaccines drove considerable hesitancy towards vaccination among the general population [34]. The inoculation policy prioritized health workers in the first wave of vaccination rollout. In some health facilities, however, health workers were hesitant to get vaccinated for fear of being harmed by the vaccines [62]. Some nurses, midwives and other allied health cadre were significantly less likely to receive the vaccines due to perceived risk of suffering from adverse reactions [37]. In some communities, people rejected the vaccines due to perceptions that they were not more efficacious than local treatment options [39]. About 70% of Ghanaians treat illness episodes including chronic conditions with traditional medicines which are believed to be efficacious and appropriate within existing cultural, religious and spiritual contexts [38, 63]. These contextual factors and misinformation combined to weaken acceptance of the vaccines.

#### Disputed second jab and change of the vaccination plan

The initial inoculation policy of the Ghana Health Service (GHS) stipulated an 8-week interval between receiving the first and second jabs. This information was communicated to enable those who would receive the first jab to prepare ahead in order to minimize default rates for the second jab. The first phase of the vaccination commenced on 1 March 2021 in designated health facilities across the country. There was assurance from the government and GHS that the second jab would be administered in time. About 7 weeks into the first jab, however, technical constraints on vaccine supply globally practically restricted the government’s efforts to have the second dose administered on schedule [43]. This prompted a critical series of disputed comments among political constituents, the public and the media about the potency of the first jab beyond the 8 weeks. The largest opposition party, for example, organized a press conference questioning modification of the dosing regimen and stating that the delay provided incentives for people to trade off the first jab or receive unauthorized jabs to remain protected [42]. Recipients of the first jab also feared being asked to repeat the vaccination process after the 8-week interval.

In the ensuing speculation, and reinforcing fierce debate within the media waves, the GHS modified the inoculation plan by extending the gap between the doses by 4 weeks. The GHS further explained that the first jab had about 76% protection for about 90 days, which coincided with the extended 12-week schedule. The extension of the dosing interval did not immediately settle public anxieties and scepticism about losing protection from the first dose. Moreover, the government’s exposition of the delay in administering the second jab was perceived as the usual politicking that characterized the COVID-19 response and management [16]. To calm anxieties, the GHS explained that about 80–94% of people who received the first jab were protected from severe symptoms and mortality associated with COVID-19 [43]. The GHS also clarified that recipients of the first jab could wait much longer if possible but cautioned against defaulting on the second jab. The second jab was eventually administered nationwide between August and September 2021 for those who received the first jab. Anecdotal evidence showed that people who experienced mild to moderate side effects such as fever, fatigue, headache and pain at the injection site from the first jab were reluctant to receive the booster [37].

#### Politically led vaccination campaign

Misinformation propagated against COVID-19 vaccine safety and efficacy in Ghana predated the vaccination rollout in the country. Misperceptions regarding the vaccines exacerbated by persistent misinformation and conspiracy theories prompted a range of interventions. In a press update to the nation on 28 February 2021, the President assured Ghanaians of the vaccine’s safety: “*I know there are still some who continue to express doubts about the vaccine, others have expressed reservations about its efficacy, with some taking sides with conspiracy theorists who believe the vaccine has been created to 'wipe out' the African race. This is far from the truth. As your President, I want to assure you that the vaccine is safe*” [44]. In clear demonstration of proof of the vaccine’s safety, the President and the Vice President launched the vaccination campaign by being the first to take the AstraZeneca COVID-19 jab on live television ostensibly to engender trust and encourage vaccine acceptance [64]. These scenes of attractive political appeal, however, did not lead to significant improvement in attitudes towards vaccination. Vaccine hesitancy in some regions led to the expiry of some 500 doses during the first wave of vaccination [39]. Another 480 doses of the AstraZeneca vaccine delivered to one of the regions were reported to have gone waste due to hesitancy [43].

To further address the broader negative social factors influencing hesitant attitudes towards vaccination, members of Parliament were encouraged to step up campaigns by highlighting vaccination as a social duty of the individual. This involved reaching out to their constituencies with clear messages about the benefits of the vaccination and taking jabs before the full view of people to change the mindset about the vaccines. In a debate on the floor of Parliament in March 2021, a member said, “*For political reasons, it is better to take the vaccines in our constituencies to inspire people to get involved in the vaccination*” [29]. Parliament also approved budget support for state institutions such as the National Commission for Civic Education (NCCE) to carry out education targeting vaccine myths and instilling trust and confidence in the vaccines.

### Actor power dynamics: stakeholders’ roles, interests and positions in the vaccination rollout

Five key stakeholders played vital roles in the COVID-19 vaccination rollout. The President, the Minister of Health, the largest opposition political party (National Democratic Congress [NDC]), the Pharmaceutical Manufacturers Association of Ghana (PMAG) and the EU were key stakeholders whose actions, interest and positions were connected to the vaccination rollout (Table 2). Following the outbreak of COVID-19 in Ghana, the President demonstrated the political leadership required to contain the pandemic [65, 66]. The President played a leading role in setting up and heading a national COVID-19 response team to mobilize the requisite political, social and economic support for mitigating the pandemic. The President used live televised speeches to highlight the government’s interventions to secure enough vaccines to break the transmission cycle [46]. The President also deepened ties with WHO, the EU and other multinational agencies for vaccine supply to Ghana.

**Table 2:** Stakeholder interest, positions and roles liked to local manufacture of COVID-19 vaccines

Stakeholder	Interest	Position	Strategies deployed	Level of influence
President of the Republic of Ghana	• Promote vaccine security • Make vaccines accessible to all • Reduce dependence on foreign vaccines • Reduce cost of vaccine procurement • Spur growth in the pharmaceutical sector • Increase employment in the pharmaceutical sector	Strongly supported the drive for locally produced vaccines	• Press briefings • Bilateral and multilateral agreements • Lobbied global leaders	Very high
Minister of Health	• Promote vaccine security • Ensure vaccines are available, accessible and affordable • Increase vaccine acceptance • Empower local pharmaceutical producers	Strongly supported the drive for local vaccines	• Press briefings • Engaged with pharmaceutical companies • Aided in the transfer of technology and waiver of patent • Chaired COVID-19-related committees	Very high
Opposition political parties	• Promote vaccine accessibility among constituents • Save lives of their constituents	• Supported the drive for local vaccines • Opposed reliance on foreign vaccines • Resisted extra taxes for vaccines	• Press statements • Parliamentary and media debates	High
Pharmaceutical Manufacturers Association of Ghana	• Expand economy of scale • Increase revenue • Increase market share • Export vaccines to African countries	• Strongly supported the drive for local vaccines • Formed a consortium seeking government support	• Media engagements • Lobbied government to implement policy for local production of vaccines	Very high
European Union	• Export technology and skill • Ensure vaccine justice	Supports the drive for local vaccines	Bilateral agreements	High

Given the global vaccine supply shortages in 2021, the President called for the production of vaccines locally to reduce dependence on Asia, Europe and North America. In an address to the Pan-African Parliament in South Africa, the President challenged African leaders to exploit opportunities for local vaccine production, noting, “*It is obvious that we need each other, and more so in combating the COVID-19 pandemic. We must develop our capacity to produce our own vaccines so that we can more effectively deal with future pandemics and not be dependent on foreign supplies and benevolence*” [67]. To achieve the vision of local vaccine production, the President established a committee to produce an action plan to guide investment policy decisions. The committee report suggested the creation of a national vaccine institute (NVI) to among others: (1) establish vaccine manufacturing plants; (2) undertake research and development; (3) forge bilateral and multilateral partnerships with vaccine manufacturers; and (4) build human capital requirements [68]. The government promised to inject seed capital of US$ 25 million of the US$ 200 funding needed to begin vaccine production by 2023 [49].

The Health Minister, on the other hand, was at the forefront of establishing agreements with vaccine manufacturers, COVAX and multinational agencies for the procurement and supply of vaccines. The Minister further led the development of a national deployment and vaccination plan (NDVP) in line with the WHO SAGE value framework for allocating and prioritizing COVID-19 vaccination rollout [47]. Innovations such as an electronic register for vaccines, virtual training platforms for health workers and vaccination apps were developed by the MoH to accelerate the vaccination rollout [47].

Political constituents and key actors in the health sector and pharmaceutical value chain supported the government’s decision for local vaccine production [48]. The opposition party, for instance, issued a statement urging the government to discontinue the western-tested vaccines and instead produce Ghana’s own patented vaccines from contextual clinical trials to increase the availability, affordability and acceptance of vaccines [42]. The PMAG comprises a 40-member association of medium- and large-scale manufacturers with a US$ 700 million market share in the pharmaceutical industry [69]. The PMAG targets 70% production of medicines to meet local needs [70] and therefore supported the move by government to boost access to vaccines through local production. The decision by the President to produce vaccines locally presented a window of opportunity for the PMAG to leverage its large market share within and across the country.

The PMAG subsequently formed a consortium to develop strategic actions to ensure a sustained, timely and uninterrupted supply of vaccines for viral and bacterial infections in the subregion [50]. Three members of the PMAG made partial payment for a vaccine manufacturing plant and approached AstraZeneca plc for waiver of intellectual property rights and technology transfer to enable them to manufacture vaccines locally [48, 68]. The PMAG, however, stressed the need for the government to create a sustainable market space and an enabling policy environment for the local vaccines to thrive. A member of the PMAG noted that: “*We need government’s commitment to purchase and a guaranteed market for the vaccines because they cannot be sold on the open market or over the counter*” [48].

The EU, one of Ghana’s main donors, expressed support for locally manufactured vaccines. The EU Vice President, for example, indicated that Ghana was considered as a possible manufacturing hub for COVID-19 vaccines in Africa [51]. A task force partnership between Ghana and the EU was set up to develop a strategic framework for vaccine production. Ghana was expected to benefit from funding from the European Investment Bank (EIB) when bilateral agreements and working arrangements were concluded [51].

### Political dynamics of the COVID-19 vaccination

#### The politics of vaccine availability

The surge in daily infections and mortalities across the country [53] was a crisis that placed COVID-19 vaccination firmly on government’s agenda [54]. The President in his series of televised speeches to the nation on the COVID-19 mitigation approaches assured Ghanaians that the government was doing everything possible to attain heard immunity by vaccinating the entire population [34]. The President also stressed that by end of 2021, about 20 million (65%) Ghanaians would be vaccinated. However, this was far from reality. As of 28 August 2021, the share of the Ghanaian population with full and partial vaccination stood at 1.3% and 2.98%, respectively [55]. Ghana was among the poorly vaccinated countries in Africa, with only four doses per 100 people administered compared with an average of 14.4 doses per 100 in the continent as of August 2021 [56].

The slow pace of vaccine acquisition and vaccination rollout drew criticisms especially from the largest opposition political party and other political elites that perceived the government to be highly political rather than tactical in dealing with vaccine supply deficits [42]. Parliament approved US$ 420 million in the 2021 budget estimates for the purchase of 42 million doses of COVID-19 vaccines at a unit cost of US$ 10. The budget allocation was well above the combined average of US$ 131.9 million budgetary estimates of Angola, Botswana, Ethiopia, Eswatini and Kenya for the purchase of vaccines [57]. Thus, the opposition party’s main argument was that government should as a matter of urgency de-risk vaccine purchase by entering into agreements with a number of manufacturers to accelerate vaccine availability. However, government felt that the issue was not about tackling risk factors at multiple points of the global supply chain, but rather that the politicization and control of the COVID-19 vaccines by the developed countries crowded out fair economic competition. Vaccine nationalism as an obstacle to vaccine supply was further highlighted in the President’s 25th update to the nation:

*International vaccine politics and the unpredictability of the supply chain, as well as a third wave of infections in some countries in Europe and Asia, have meant that we have not been able to secure as many vaccines and vaccinated as many Ghanaians as we would have wanted at this stage.* [71]

The government’s position about vaccine nationalism as a threat to vaccine procurement was keenly contested by opposition parties challenging the government to be proactive and swift in securing enough vaccines in the shortest possible time to save lives [42]. During a debate in the chamber of Parliament, the majority side laid emphasis on the President’s lamentation about vaccine nationalism as a barrier to securing sufficient vaccines. The minority, however, demanded that the government move beyond the rhetoric and craft a vision that would propel Ghana to secure more vaccines and deliver the citizens from the grip of COVID-19.

#### The politics of vaccine procurement

Rising mortality rates associated with the pandemic invoked panicked responses by governments globally. The speed to procure vaccines to save lives and reduce suffering created avenues for corrupt behaviours and shoddy deals in some countries [59, 60]. But elements of procurement corruption, procurement breaches and irregularities in the vaccine distribution chain were not completely detached from politics. In Ghana, the political and civil pressure to secure sufficient supply of vaccines led to procurement irregularities. On 9 March 2021, Ghana’s MoH entered into a procurement contract with a United Arab Emirates royal and a private company, S.L. Global, for the supply of 3.4 million doses of the Sputnik V COVID-19 vaccine at a unit cost of US$ 19 [35]. The decision to procure each dose at almost double the factory price of US$ 10 attracted backlash and criticism from the media, opposition political parties and civil society organizations. Sections of the public also questioned the Ministry’s decision to use middlemen to procure overpriced vaccines.

The litany of public comments also pointed to lack of due diligence in the procurement process. The government responded that the procurement process was prudent and well scrutinized and that it negotiated the unit cost down to US$ 19 from an initial quoted US$ 25. The government also argued that the supplier factored in additional ex-factory costs relating to shipment, land transport, handling and special storage charges in reaching the deal [32]. Amidst the controversies and the public outcry, a nine-member parliamentary ad hoc committee was convened to investigate the procurement transaction. Among other findings, the committee investigation revealed that: (1) the transaction received neither parliamentary nor cabinet approval as required by relevant statutory laws; (2) middlemen were used to procure and distribute vaccines, which is improper; and (3) the procurement process was characterized by lack of due diligence and violations of relevant procurement legislations [35]. The committee tasked the Minister of Finance with taking steps to recover the 50% (US$ 2.85 million) partial payment of the contract sum that had already been made to the supplier.

Based on the committee’s report, the media, opposition political party and other political constituents labelled the procurement deal as fraudulent and shady. Moreover, the Health Minister was profoundly criticized for committing a “political sin” given the lack of professionalism in handling the procurement deal. Sections of the public also called for his “head” [35]. A pressure group called OccupyGhana, for example, organized a press conference demanding resignation of the Health Minister [72]. The Minister, however, resisted any attempt to remove him from office, insisting that his thoughts and actions were blurred by the abnormalities of the time. The Minister also asserted that the procurement contract was necessitated by failed systematic diplomatic attempts to secure vaccine deals directly from the Russian manufacturer. The deal turned dramatic when the supplier defaulted in meeting several deadlines, while the Minister felt unable to terminate the deal, citing frustration in finding alternative suppliers. The President in a press update to the nation on 2 May 2021 assured Ghanaians of adequate Sputnik V vaccine supply [34]. However, the complexities and cynicism that characterized the Sputnik V procurement deal somewhat stifled the political effect of the President’s drive to meet vaccine needs. At the time of the study, the deal was terminated and the Health Minister had received political backing to stay in office, while the supplier agreed to refund the 50% down payment [35].

## Discussion

This paper drew on a policy analysis framework to understand the framing, issue characteristics, actor power dynamics and political context of the COVID-19 vaccination process in Ghana. We found that the vaccination rollout was mainly framed along the dimensions of public health, gender-centredness and UHC. These frames highlight the varied understanding or interpretations giving to the vaccination rollout. Multiple framing of the vaccination rollout can be both beneficial and detrimental. The different lenses of the vaccination ensure that different pathways of policy responses and interventions are implemented across various sectors and institutions to widen the vaccination net. Additionally, varied frames promote a range of discussion on feasible and sustainable solutions within which to prioritize policy and investment decisions on scaling up vaccination [73]. On the contrary, multiple interpretations potentially distort policy coherence and strategic direction for vaccination. Framing vaccination from a gender perspective, for example, creates incentives for targeting vaccination of women while overlooking the broader impact of vaccination on populations adversely affected by the pandemic [26, 74]. Moreover, because different frames appeal to different interests, it might be technically difficult to harmonize conflicting and competing interests among stakeholders and institutions seeking to advance the course of vaccination scale-up.

The key issue characteristics surrounding the vaccination rollout were vaccine hesitancy, contestations around delayed second jab, and political actors driving the vaccination campaign. Vaccine hesitancy was shown to be driven by a combination of factors. First, and in consonance with earlier studies, was a lack of trust or doubt about the vaccine safety and potency [8, 75]. Distrust shaped by individual and societal beliefs, values, norms and perceptions regarding COVID-19 management have been shown to contribute to hesitancy for vaccination [37]. Second, vaccine hesitancy was partly the result of an infodemic disseminated across social media channels portraying the vaccines as harmful for human consumption [76]. This finding supports studies in other countries attributing vaccine hesitancy to the spread of misinformation and inconsistent scientific facts regarding the vaccines [77, 78]. Third, vaccine hesitancy was associated with beliefs that traditional medicines are more efficacious for the treatment of COVID-19. This finding reinforces the conspiracy narrative and beliefs elevating traditional therapeutic approaches above vaccine treatment of COVID-19 [79, 80].

Different stakeholders were found to have differing domains of interest and positions in the COVID-19 vaccination rollout. These interests and positions were connected to the decision by the President to promote local production of COVID-19 vaccines to address vaccine supply deficits. However, there appeared to be commercial and profiteering motives underlying the PMAG support for the proposed local production of vaccines. Such motives were driven by the fact that Ghana is largely an import-dependent retail pharmaceutical market with low prices of foreign medicines [81]. As local pharmaceutical firms struggle to compete favourably with their foreign counterparts, the PMAG envisaged local production of COVID-19 vaccines as an opportunity to widen their market space and increase profit margins through an increased product portfolio.

Furthermore, the findings correspond with existing studies showing that COVID-19 vaccine manufacturing, acquisition and vaccination rollout are driven substantially by politics and political behaviours [59, 82, 83]. Political behaviours and political contentions regarding the vaccination rollout reinforce the politicization and political polarization that characterize government interventions, financing and management of the COVID-19 pandemic in Ghana [13]. The government response pattern and mitigation approaches to the COVID-19 pandemic were almost always subject to bipartisan political interpretations shaped by underlying political beliefs and ideological positions. For instance, while the government’s lockdown mitigation measures such as the provision of free meals and water, electricity subsidies and tax waiver for health workers were perceived as helpful [14, 84], the rival political party interpreted such interventions as populist appeals to win votes in the 2020 elections [40]. Thus, for the opposition party, the snail pace of the vaccination rollout amid assurance by the President of achieving herd immunity in the short term portrayed populist rhetoric to soothe the public [42]. Political sentiments and differences on vaccine supply and vaccination coverage were not peculiar to Ghana. Evidence pointed to political constituents employing blame game tactics and dramatization of the pandemic to attract government attention to supply vaccines [85].

The politics of vaccine procurement and the circumstances underlying the shoddy deals align with reports in the literature [60]. Just like other LMICs without the requisite financial strength to procure directly from vaccine developers, Ghana subscribed as an advance market recipient to take vaccines from the COVAX pool procurement facility. However, vaccine nationalism that resulted in vaccine hoarding, stockpiling, export bans and profiteering motives weakened COVAX’s capacity to secure enough vaccines for equitable distribution [86]. In response, LMICs resorted to the private supply chain market to procure vaccines. Limited regulation of the operation and pricing regime of private markets created vulnerability to procurement lapses as shown in this study. Kohler and Wright [87] noted that COVID-19 vaccine procurement irregularities such as those identified in this study were to be expected given the lack of transparency and proper accountability in the bidding and selection of suppliers. This situation was exacerbated by the high level of political control and sole-sourcing mechanisms of vaccine procurement, leaving limited room for proper scrutiny, accountability and value for money [60, 88].

This study has some limitations to acknowledge. We used a rapid review approach to give a quick highlight of the policy dynamics of the COVID-19 vaccination rollout, using data from media reports, journal articles and other publicly available documents. The rapid review approach limited the inclusion of a wide range of scientific literature sources that could provide more in-depth information on the topic. Moreover, our inability to triangulate the findings from this rapid review with perspectives from key informant interviews means that the veracity of some of the claims from the media and other public sources cannot be fully established. Despite these limitations, however, we believe that the findings of this study are reflective of the policy dynamics of the early phase of the COVID-19 vaccination rollout in Ghana. We also acknowledge that emerging developments of COVID-19 and changing dynamics in subsequent phases of the vaccination rollout demand caution in generalizing these findings beyond the study context and time frame.

## Conclusion

This rapid review demonstrates Ghana’s struggle to keep pace with the promising start of the COVID-19 vaccination. We have shown how multiple interpretations and political contentions intertwined with different stakeholder interests and positions along with adverse perceptions to shape the vaccination rollout. Scaling up the vaccination would require a combination of efforts including: (1) taking steps to harmonize the different frames with potential negative effects on policy coherence; (2) securing political unity and uniformity of ideas as well as political participation in creating structures for sustainable vaccine acquisition and vaccination; (3) harnessing support from stakeholders in the private sector to increase vaccine availability and accessibility; and (4) tackling the complex web of social factors embedded within the media and community structures that adversely shape perceptions and acceptance of vaccines. Finally, to promote inclusive vaccination, the government should extend vaccine education and vaccination sites to peripheral settings to reduce the level of anxiety and fear limiting vaccination uptake.

## Data Availability

All documents reviewed for this study are already publicly available.
